# Fabrication of Pressure Conductive Silicone Rubber Socket Device by Shape-Controlled Nickel Powders Produced by High-Energy Ball Milling

**DOI:** 10.3390/ma15196670

**Published:** 2022-09-26

**Authors:** Maddipatla Reddyprakash, Daseul Kim, Woo-Jeong Choi, Ji-Hyeon Yun, Chadrasekhar Loka, Kee-Sun Lee

**Affiliations:** 1 Department of Advanced Materials Engineering, Kongju National University, Cheonan 31080, Korea; 2 TFE, Ltd., 50-8, Banwol-gil, Hawsung-si 18384, Korea

**Keywords:** silicone rubber socket, shape-controlling, flake-type Ni, magnetization, high-energy ball milling

## Abstract

The pressure conductive silicone rubber socket (PCR) is one of the promising test socket devices in high-speed testing environments. In this study, we report highly dense PCR device channels comprised of high aspect-ratio flake-shaped Ni powders. The shape-controlled Ni powders are prepared by the high-energy milling process. The scanning electron microscopy (SEM) and particle size analyzer (PSA) results of the synthesized powder samples showed well-defined flake type Ni powder morphology, and the powder sizes are distributed in the range of ~24–49 μm. The cross-sectional SEM study of the fabricated PCR revealed that the channels consisting of flake Ni powder are uniformly, densely distributed, and connected as face-to-face contact. The resistance of the PCR channels comprised of flake-shaped Ni powders showed ~23% lower resistance values than the spherical-shaped Ni powders-based channels, which could be due to the face-to-face contact of the powders in the channels. The magnetic properties study for the flake-type Ni powder showed a high remanence (~2.2 emu/g) and coercivity (~5.24 mT), owing to the shape anisotropy factor. Finally, the fabricated highly dense and conductive channels of the silicone rubber socket device by shape-controlled Ni powder could be a potential test socket device.

## 1. Introduction

Demands for new electronic devices with higher operating speeds have been steadily increasing in the field of high-speed communication networks, mobile phones, autonomous cars, and the artificial intelligence industry. Therefore, high-performance packages should fortify the high data rates. Low-power double data rate (LPDDR) technologies require a drastic increase in the amount of processing data [1,2]. Nevertheless, testing such high-performance semiconductor integrated circuits (ICs) is also equally important to not only distinguish the defective product but also to ensure the quality of the device. To conduct the test for the high-performance packaged ICs, soldering is a usual method for the vertical interconnections between the device under test (DUT) and the printed circuit board (PCB) [1,3,4]. Despite this, it has certain limitations, such as the damage to the semiconductor devices and PCB pads due to the hard contact (high contact force), poor electrical performance with partial current loss, applicability in smallest pitch test environments, and reusability. In this case, as the representative test methods, pogo- and silicone rubber-typed pressure conductive socket devices were adopted, which can be reused over tens of thousands of repetitive tests [5,6]. They will act as a mediator for current transportation through the physical contact between the solder ball of the packaged chip (ball grid array) and PCB, which are installed in the compression system (handler) [7,8,9]. In the case of the pogo-type socket device, the conducting channels are comprised of a long spiral metal spring, and the interference between the narrow current channels causes severe noise and frequency-dependent insertion loss (inherent parasitic components) [10,11]. In addition, due to the oxide layer or contamination during the contact between the pogo-tip and the package ball, strong pressure should be applied to penetrate the layer, which will eventually damage the tip as well as the ball [12]. In order to overcome the aforementioned limitations, the pressure conductive silicone rubber socket (PCR), an elastomer-type socket comprised of silicone-rubber and metal powders that becomes electrically conductive when the socket is under compression, has gained increasing attention. The PCR allows the fabrication of a high-density test socket and offers superior electrical properties because of its short electron transport (channel) length compared with the pogo pin socket [13,14]. Therefore, the PCR has been emerging as a high-frequency operational socket device. Precious metals and the metal-alloys such as Ag, Au, AuNi, AuCo, SnPb, and PdNiAu have been reported for the socket [12,15,16,17]; however, they have certain limitations such as high costs and complicated synthesis routes. Nevertheless, nickel (Ni) is a well-known ferromagnetic material with high electrical conductivity and corrosion resistance even at elevated temperatures, which can form an alloy with other metals. As a result, most of the researchers exploited the Ni powder (spherical or near-spherical) for the PCR channels, which results in poor electrical conductivity due to the point contact [1,3]. Despite this, fabrication of PCR with the shape-controlled Ni powders was seldomly reported. Accordingly, in this work, the technical transition from point contact to face contact between powders is tried through flake-shaped Ni powders, indicating improved conductivity.

Horizontal-type high-energy ball milling is an effective method for the mass-scale production of micron-sized powder [18]. In addition, the studies reported that horizontal-type milling is advantageous to accomplish the homogeneous distribution of the powder as compared to vertical-type milling systems such as the attritor milling system in which the powder segregation occurs (dead-zone formation) due to the gravity effect [19]. The evolution of microstructure could be controlled by the milling parameters, especially the milling speed [20]. In this study, the flake-shaped Ni powders are produced through a horizontal-type high-energy ball milling process. The influence of the milling environment on the size and morphological changes of the Ni powder is investigated. Based on Hertzian mechanics, the deformation mechanism of the Ni powder is discussed. The produced powders are fabricated into a silicone rubber-based socket device in response to the externally applied magnetic field. The electrical properties of the prepared socket device are studied. The densification of Ni powders and shape change of the channels are investigated. Finally, the magnetization versus applied magnetic field hysteresis loops for the prepared Ni powders is studied at room temperature.

## 2. Experimental

### 2.1. Synthesis of the Flake Type Ni Powder

The spherical shape Ni powder with a size range of 16–20 μm (Ntrium Co., Hwaseong-si, Korea) is used as a starting powder for the preparation of flake shape (high aspect-ratio) Ni powder. The powders are mechanically milled by using a horizontal type high-energy mechanical milling (HEM) machine (Simoloyer CM01, Zoz GmnH, Wenden, Germany) for 30 min with a milling ball-to-powder weight ratio of 10:1. The silicon nitride (Si_3_N_4_) balls with a diameter of 3 mm are used for milling the powder. The milling was conducted by the addition of 8 wt% of ethanol as a processing controlling agent (PCA). The milling was carried out at different rotation speeds (@500, 700, and 1000 rpm) of the impeller (spindle).

### 2.2. Socket Device Fabrication

**Figure 1:**
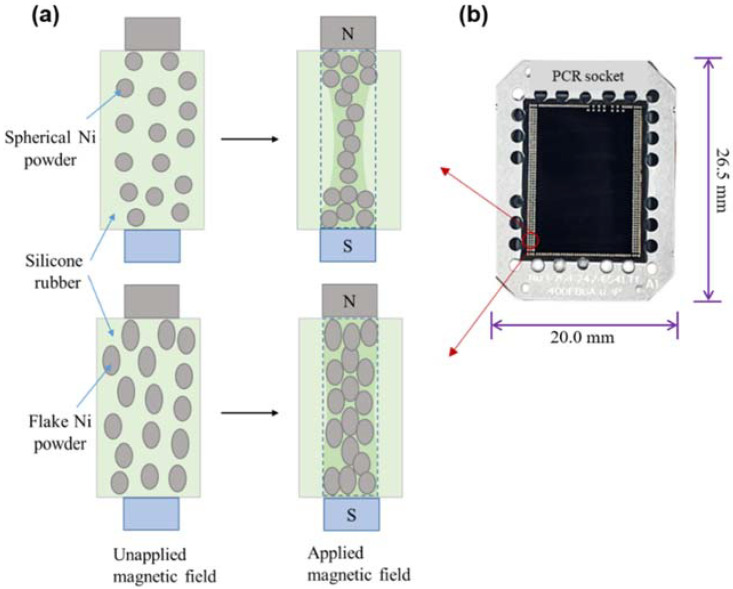
(**a**) Schematic diagram of the fabrication process of the pressure conductive silicone rubber socket device and (**b**) photograph of the prepared PCR socket device.

The PCR is comprised of conductive Ni powders embedded in silicone rubber composite. Figure 1a illustrates the schematic diagram of the fabrication process of the PCR device. First, the Ni (spherical or flake shape) powder with 20 wt.% is mixed with liquid silicone rubber and a hardening agent (1:1) using a liquid silicone rubber mixing machine (Model: VMX-N360, EMC Co., Seoul, Korea) to form the slurry. Then, the slurry was poured into a socket mold, which was loaded into the electromagnet silicone socket forming machine (the magnetic forming machine). When the magnetic field was not applied to the slurry (i.e., the mixture of Ni powder, liquid silicone rubber, and hardening agent), the Ni powder was randomly dispersed in the liquid silicon rubber matrix. After applying the magnetic field (1.5 Tesla) to the slurry, the Ni powder was aligned or oriented parallel direction to the applied magnetic field. Subsequently, the curing temperature (120 °C) was applied to the slurry. Thus, the pressure conductive silicone rubber socket (20.0 mm × 26.5 mm) comprised of cylindrical shaped conductive channels (diameter: 200 μm) of pitch size ~300 μm was fabricated (Figure 1b).

### 2.3. Characterization

The powder morphology of the as-synthesized samples was analyzed by using scanning electron microscopy (SEM; MIRAH, TESCAN). The particle size and distribution were analyzed by a particle size analyzer (PSA; SALD-2300, SHIMADZU). The cross-sectional and surface images of the fabricated silicone rubber socket devices are observed by SEM, and the relevant specimens were prepared using focused ion beam (FIB) milling. The surface area packing factor of the distributed Ni powder (spherical and flake-type Ni) in the socket device is estimated by ImageJ software. The surface area packing factor of the Ni powders in a channel was calculated by A_P_/A_C_, based on the channel dimension (defined area, (A_C_)) and the cross-section area of the Ni powders (A_P_) in a channel using ImageJ software.

Magnetization (M) vs. the applied magnetic field (H) curves for the spherical and flake type Ni powders were recorded by a vibrating sample magnetometer (VSM) at room temperature. The electrical resistance of the socket device was measured by using a socket device electrical resistance test with a contact force of 20 g/pin.

## 3. Results and Discussion

### 3.1. Mechanical Milling-Driven Microstructure Evolution of Ni Powder

**Figure 2:**
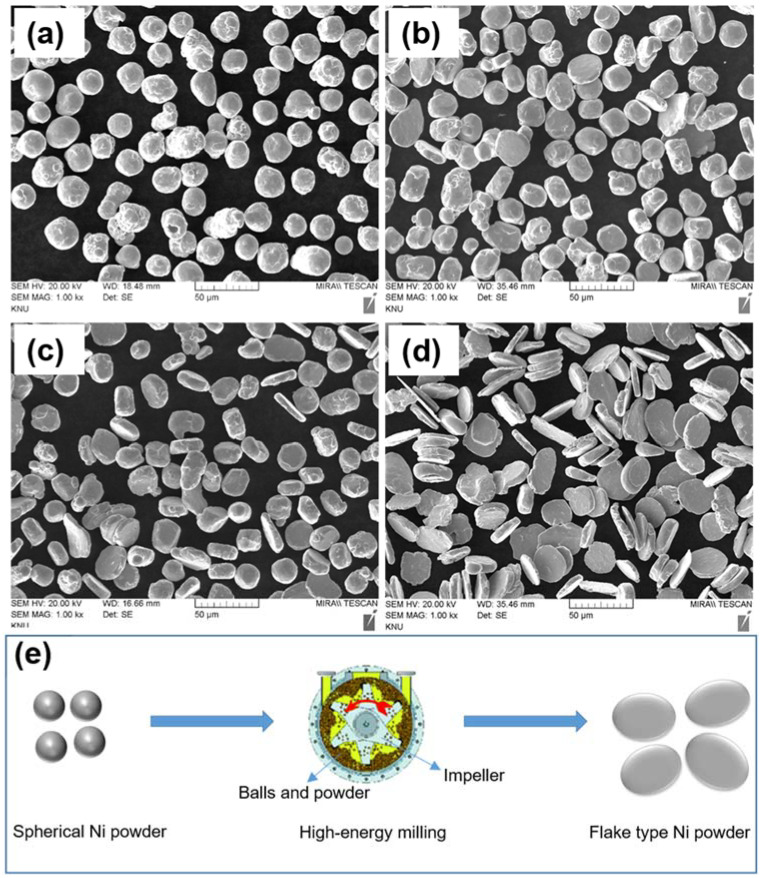
Scanning electron microscopy images of (**a**) pristine Ni powder; Ni powder milled for 30 min at a rotation speed of (**b**) 500 rpm, (**c**) 700 rpm, (**d**) 1000 rpm, and (**e**) the schematic illustration of the synthesis of the flake-type Ni powder.

Morphologies of the pristine Ni and as-milled Ni powders at different rotation speeds (500–1000 rpm) were observed by SEM. As presented in Figure 2a, the pristine Ni powders are a spherical shape morphology with sizes ranging from 15 to 20 μm. Figure 2b shows the morphology of the mechanically milled precursor spherical Ni powders for 30 min at the rotation speed of 500 rpm. Compared with pristine Ni powder, the milled powders are in various shapes of distorted/elongated and flake along with un-milled spherical powders. Very few of the powder shapes were changed to the flake shape. Thus, the majority of unchanged Ni powder shapes at 500 rpm indicate that the deformation energy induced by the ball milling process could not be sufficient. At 700 rpm (Figure 2c), the powder particles showed mixed shape morphology, such as flake and spherical shape. However, the majority of the powder is comprised of flake shape as compared with 500 rpm condition. Each Ni flake powder thickness is ~8 μm, and the average diameter is ~20–32 μm. In the case of 1000 rpm (Figure 2d), it can be observed that the spherical morphology of the starting Ni powder was completely changed to the flake-type and smooth morphology. Moreover, the flake-type powder is not cladded or agglomerated, which is due to the addition of the processing control agent. The average thickness of the flake-type is determined to be ~5μm, and the diameter is ~20 to 45 μm. The SEM images revealed that the critical rotation speed to transform the spherical shape powders into flake-type Ni powders is 700 rpm. Figure 2e shows the schematic illustration of the preparation of the flake-type Ni powder.

**Figure 3:**
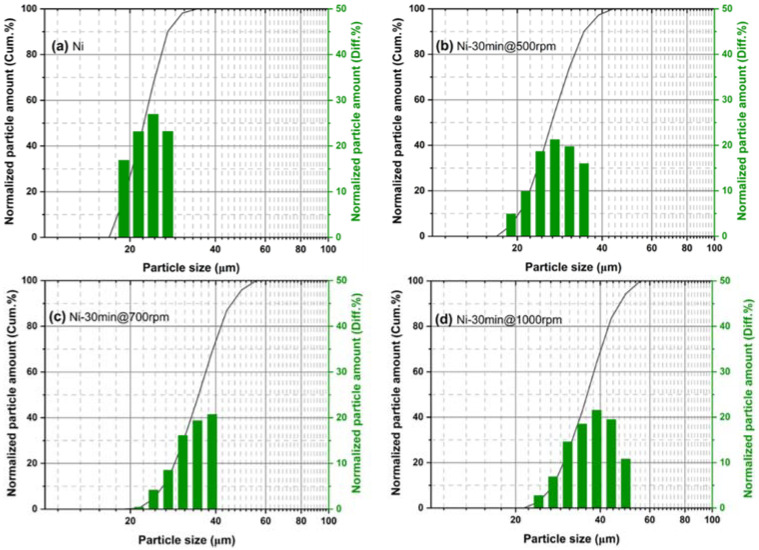
The particle size distribution of (**a**) pristine Ni powder and as-milled Ni powder for 30 min at (**b**) 500 rpm, (**c**) 700 rpm, and (**d**) 1000 rpm.

The particle size distribution of the as-received Ni powder and milled Ni powders is presented in Figure 3. As displayed in Figure 3a, the particle size distribution of the as-received Ni powder is distributed in the range of 15–25 μm, and the median particle size is about 22.7 μm. Figure 3b illustrates the particle size distribution of the high-energy mechanical Ni powder for 30 min at a rotation speed of 500 rpm. The powder sizes are distributed in the range from 16 to 32 μm, with a median particle size of ~24.13 μm. At 700 rpm for 30 min of milled Ni powder (Figure 3c), the particle sizes are distributed, ranging from 21 to 39 μm, and the median particle size is about 29 μm. At 1000 rpm for 30 min condition, the milled Ni powder (Figure 3d) sizes are dispersed in the range from 24 to 49 μm. The median particle size is about 34.5 μm. The rotation speed is one of the important milling parameters for controlling final powder size [21]. Thus, the particle size analysis revealed that at the lowest rotation speed (i.e., 500 rpm), the Ni powder sizes were not significantly changed compared with the as-received spherical powder, demonstrating that the deformation energy prompted by the ball milling process is not sufficient. As the rotation speed increases from 500 to 700 and 1000 rpm, the powder size distribution is also shifted from smaller size to larger size, which indicates that Ni powder gets flattened and has a plastic deformation effect. Consequently, the well-defined flake-type Ni powders could be obtained at 1000 rpm. The produced flake-type Ni powders were utilized for the fabrication of the pressure conductive silicone rubber socket device.

### 3.2. Milling Mode and Deformation Mechanism of Ni Powder

**Figure 4:**
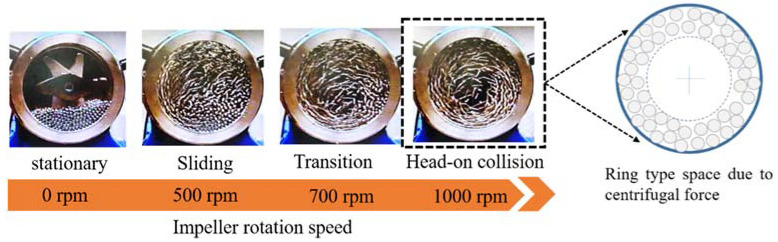
Ball-to-ball collision mode changes depending on the rotation speed of the impeller.

The collision of milling balls in the high-energy (speed) milling system can be changed from the shearing (sliding, friction) mode to head-on collision mode with an increase in the rotation speed of the impeller (Figure 4). In the case of 500 rpm, milling balls are partially rotated along the chamber wall and stagnant on the bottom side of the chamber due to the low centrifugal force leading to the shearing mode, which is expected to be ineffective in accomplishing homogeneous energy transfer. However, in the case of 1000 rpm, most of the milling balls are rotated in the ring-type space of the chamber wall. As a result, the head-on collision mode is achieved in the ring-type space of the milling chamber leading to homogeneous energy transfer to the Ni powders.

**Figure 5:**
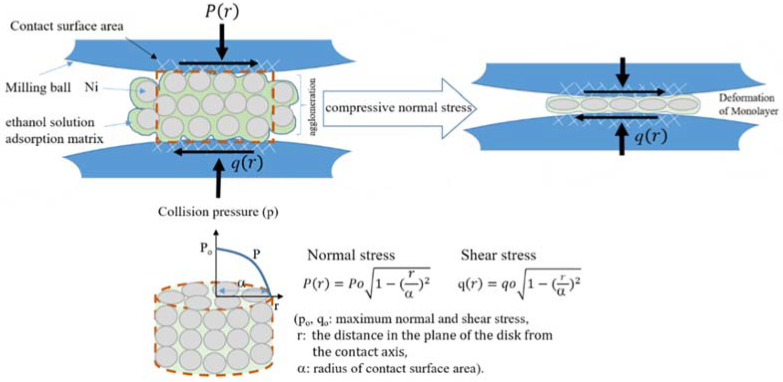
Schematic diagram showing the stress transfer over hypothetical Ni-powder agglomeration between the collision balls based on the modified Hertzian mechanics.

Based on Hertzian mechanics, assuming that the Ni powders are agglomerated in a cylinder (bundled powders) shape due to the absorption of ethanol solution, in head-on collision mode of milling condition, normal stress (P(r)) and shear stress (q(r)) apply to the surface of cylinder-shaped agglomeration [22,23] of Figure 5. In this case, shear stress (q(r)) can be assumed to be negligible, which is due to the reduced friction driven by the absorption of ethanol solution between the milling (collision) balls and the surface of a cylinder. As a result, normal stress (P(r)) is just applied to a hypothetical cylinder comprised of Ni-powder agglomeration, in which P(r) is similar to P_o,_ indicating that P(r) is uniformly applied to the contact surface of the Ni-powder agglomeration since the radius of contact surface area (α) is enough larger than that of the cylinder. As normal stress (P(r)) applies to the cylinder, the Ni powders in agglomeration are taken apart by the sliding motion of powders and form the monolayer between the milling balls, which continue to be plastically deformed into the flake shape. Since the radius of curvature of the milling ball is far greater than that of the Ni powder, a significant number of powders are expected to be placed under the normal stress condition. Thus, the produced powders were observed to be flake shape without cladding or agglomeration, which was well consistent with the above theoretical analysis.

### 3.3. Ni Powder-Based Pressure Conductive Silicone Rubber Socket Device

**Figure 6:**
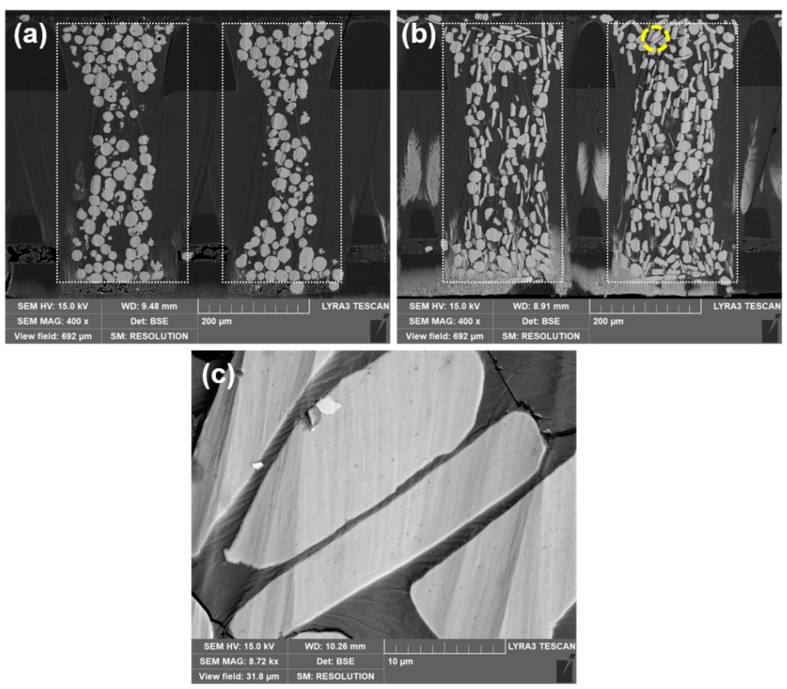
The cross-sectional scanning electron microscopy images of the fabricated pressure conductive silicone rubber socket device with (**a**) spherical Ni powder, (**b**) flake-type Ni powder: dotted lines are for defining the channel area, and (**c**) high-magnification image of flake-type Ni powder (encircled area).

In the process of PCR device fabrication, the application of a magnetic field is a prerequisite to ensure well-defined channels. The cross-sectional SEM images of the prepared PCR are presented in Figure 6. The cross-sectional SEM image of the socket device fabricated with spherical-shaped Ni powder (Figure 6a) showed that each channel was surrounded by a silicone rubber matrix. The spherical Ni powders were non-uniformly dispersed in each channel, and they were connected from point to point. At the top and bottom regions of each channel, the powders were agglomerated and densely packed, which is likely due to the small difference in magnetic anisotropy factor of spherical shape powders. The magnetic field can be concentrated on both sides (top and bottom) of the magnetic pole. In the middle region of the channel, a relatively smaller amount of powder was distributed. As a result, the channel shape was formed to be curved. Nevertheless, in the case of socket device prepared with the flake shape Ni powders (Figure 6b), the distribution of the powder in the channels was greatly improved, which could be due to the face-to-face contact as confirmed by the high-magnification cross-sectional microstructure observation of the PCR (Figure 6c). Compared to the spherical Ni powders, the flake-type powders can be aligned to a specific direction with higher magnetic anisotropy, leading to face-to-face contact with the extended contact area.

The surface area packing factor (A_P_/A_C_) of the distributed Ni powders (spherical and flake-type Ni) in the channels of the socket device is estimated by ImageJ software. The obtained A_P_/A_C_ for the respective socket devices are 31% (channels with spherical Ni powder) and 43% (channels with flake Ni), which is presumed to be due to the preferential rearrangement of flake Ni powders in the viscous silicone rubber matrix, parallel to the applied magnetic field.

**Figure 7:**
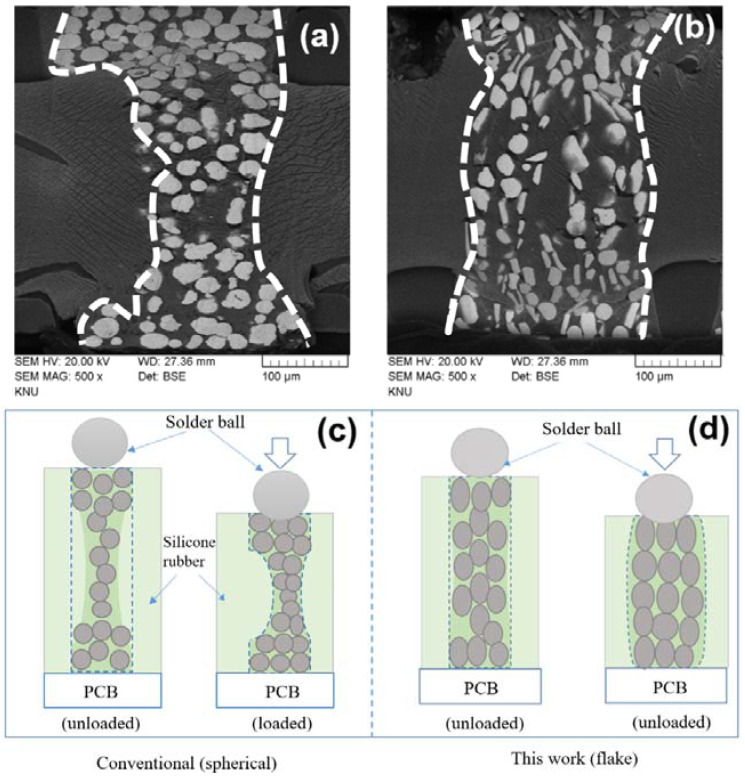
Cross-sectional SEM images of the socket channel shape change according to the compressive force of the solder ball. Socket devices with (**a**) spherical Ni powder, (**b**) flake Ni powder, (**c**,**d**) are schematic illustrations of the socket device channel deflections.

As displayed in Figure 7a, when the compressive force of the solder ball is applied over the socket device channel comprised of spherical powders, then the channel has been deflected (to the one side) from the center region, indicating the concave shape. Under the unidirectional stress condition, the channel was deflected (distorted) to the one side, as shown in Figure 7a (schematic illustration in Figure 7c), which could be due to the uneven distribution of powder (non-uniform elastic behavior) at the center region of the channel. On the other hand, the socket device channel consisting of flake-type powders (Figure 7b) revealed a relatively uniform elastic deformation, which is in the radial direction to the applied force due to the good distribution of flake powders in the channel. Figure 7c,d are the schematic diagrams of the socket device deflection under the non-contact and contact mode of the solder ball.

### 3.4. Magnetic Properties of the Ni Powder

**Figure 8:**
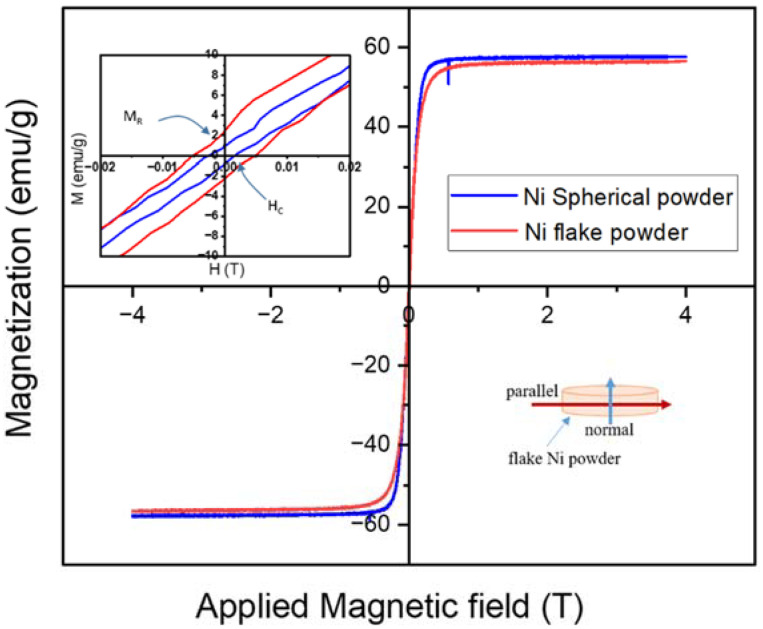
Magnetization (M) vs. applied magnetic field (H) curves for Ni spherical and flake powders at room temperature.

As shown in Figure 8, the spherical and flake-type Ni powders exhibit the same magnetic saturation (M_S_) values. The M_S_ value for the spherical and flake Ni powders are ~57.5 and ~56.7 emu/g, respectively. The inset in the Figure reveals that there is a significant change in the remanence (M_R_) and coercivity (H_C_) values. The M_R_ and H_C_ values for the spherical Ni powders are ~1 emu/g and ~2.6 mT, respectively. In the case of the flake Ni powders, the M_R_ and H_C_ values increased to ~2.2 emu/g and ~5.24 mT, which are about 220% and 200%. The changes in M_R_ and H_C_ values could be explained by taking into account the shape anisotropy factor. For spherical-shaped (polycrystalline) Ni powder with random orientation, an applied field induces a similar magnetization in any direction. On the other hand, in the case of non-spherical shape, the easy magnetization is along with the long axis rather than the short axis, which is based on the fact that the demagnetizing field along the short axis is stronger than that of the long axis direction [24]. These results are consistent with the rise in M_R_ and H_C_ values, as shown in Figure 8 (insert). Therefore, the high aspect-ratio (flake-type) Ni powder obtained from optimized milling parameters was effective in producing the high packing factor socket device by controlling Ni powder shape-dependent demagnetization factors.

The electrical resistance of the socket device channels comprised of flake Ni powder showed 30.70 mΩ compared to spherical powder-based channel resistance of 39.74 mΩ. It is the reduced resistance of ~23%, which is presumed to be due to the face-to-face contact and enhanced surface area packing factor of the flake Ni powders in the channels. For commercialized PCR, electroplating silver and gold over the flake Ni powder is required to reduce the electrical resistance. Despite the synthesized flake-type Ni powders exhibiting remarkable performance for PCR, the channel resistance can be further decreased through changes in the morphology of the powders. Thus, the present study enables Ni powder shape-dependent improved channel performance of the silicone rubber socket devices.

## 4. Conclusions

In summary, we have successfully fabricated a pressure conductive silicone rubber socket device with high aspect-ratio (flake-type) Ni powders. In this work, primarily, the high aspect-ratio flake-type Ni powders were produced through a facile and cost-effective method of high-energy mechanical milling of elemental spherical-shape Ni powder. The effect of milling speed (i.e., rotation speed) on the powder morphology and size change was studied by SEM and PSA. The SEM and PSA results of the synthesized powder samples revealed that as the rotation speed changes from 500 rpm (shearing collision mode) to 1000 rpm (head-on collision mode), then the spherical shape Ni powders are completely transformed into flake-shaped Ni powder with relatively a uniform microstructure and size is about 34.5 μm. The cross-sectional SEM image analysis of the fabricated silicone rubber socket device revealed that the high aspect-ratio flake-shaped Ni powders in the channels of the socket device are uniformly and densely dispersed and connected as face-to-face contact. However, in the case of spherical powder-based socket channels, the powders are non-uniformly dispersed in the channels, and they were connected as point-to-point contact. The electrical resistance of the socket device channels comprised of flake Ni powder showed about 23% low resistance than the spherical powder-based channels, which is presumed to be due to the face-to-face contact and enhanced A_P_/A_C_ of the flake Ni powders in the channels. The magnetic property measurements demonstrated that the flake-shaped Ni powder exhibited a high remanence (M_R_) of ~2.2 emu/g and coercivity (H_C_) of ~5.24 mT, which is owing to the shape anisotropy factor. Although the high aspect-ratio shape controlled Ni powder synthesized by varying the milling speed could be a potential candidate for making highly dense and conductive channels of the silicone rubber socket devices, further studies are required to demonstrate the shape control depending on the milling time.

## Data Availability

Not applicable.
